# Edible Leafy Plants from Mexico as Sources of Antioxidant Compounds, and Their Nutritional, Nutraceutical and Antimicrobial Potential: A Review

**DOI:** 10.3390/antiox9060541

**Published:** 2020-06-20

**Authors:** Lourdes Mateos-Maces, José Luis Chávez-Servia, Araceli Minerva Vera-Guzmán, Elia Nora Aquino-Bolaños, Jimena E. Alba-Jiménez, Bethsabe Belem Villagómez-González

**Affiliations:** 1 Recursos Genéticos y Productividad-Genética, Colegio de Posgraduados, Carr. México-Texcoco Km. 36.5, Montecillo, Texcoco 56230, Mexico; lourdes.mateos@colpos.mx; 2 CIIDIR-Oaxaca, Instituto Politécnico Nacional, Ciudad de México 07738, Mexico; avera@ipn.mx (A.M.V.-G.); bvillagonez1600@alumno.ipn.mx (B.B.V.-G.); 3 Centro de Investigación y Desarrollo de Alimentos, Universidad Veracruzana, Xalapa-Enríquez 1090, Mexico; eliaquino@uv.mx; 4 CONACyT-Centro de Investigación y Desarrollo de Alimentos, Universidad Veracruzana, Xalapa-Enríquez 1090, Mexico; jimalba@uv.mx

**Keywords:** indigenous knowledge, bioactive compounds, malnutrition, wild edible plants, dietary diversification

## Abstract

A review of indigenous Mexican plants with edible stems and leaves and their nutritional and nutraceutical potential was conducted, complemented by the authors’ experiences. In Mexico, more than 250 species with edible stems, leaves, vines and flowers, known as “*quelites*,” are collected or are cultivated and consumed. The assessment of the quelite composition depends on the chemical characteristics of the compounds being evaluated; the protein quality is a direct function of the amino acid content, which is evaluated by high-performance liquid chromatography (HPLC), and the contribution of minerals is evaluated by atomic absorption spectrometry, inductively coupled plasma-optical emission spectrometry (ICP-OES) or ICP mass spectrometry. The total contents of phenols, flavonoids, carotenoids, saponins and other general compounds have been analyzed using UV-vis spectrophotometry and by HPLC. For the determination of specific compounds such as phenolic compounds, flavonoids, organic acids and other profiles, it is recommended to use HPLC-DAD, UHPLC-DAD, UFLC-PDA or gas chromatography-mass spectrometry. The current biochemical analysis and biological evaluations were performed to understand the mechanisms of action that lead to decreased glucose levels and lipid peroxidation, increased hypoglycemic and antitumor activity, immune system improvement, increased antibacterial and antifungal activity and, in some cases, anti-Helicobacter pylori activity.

## 1. Introduction

Diets in all countries are essentially based on the production of a few cultivated species—corn, rice, wheat, potato, soybean, barley, various vegetable species, yuca, beet, tomato, banana and watermelon—and nearly 160 edible species with worldwide distribution have been recorded [1]. The predominance of a few cultivated species and products in the market leads to increased dietary homogeneity; there is a direct relationship between a high diversity of natural edible products in supply systems and dietary variety, which influences the nutritional quality of the diet and affects the health status of individuals. Additional issues include transitions in dietary patterns, the Westernization of diets and the increased consumption of foods of animal origin, foods containing refined flour and sugar or foods with high caloric value. A low-quality diet increases the risk of undernutrition and malnutrition, and this is exacerbated by socioeconomic, cultural and political factors such as income, food education, an increased supply of edible products without nutritional value, inequity, access to food, cultural identity and gender issues [2,3,4].

Dietary diversification is an essential principle for optimal human nutrition. For example, a diet that is diverse in sources of minerals, amino acids and proteins, vitamins, vegetable fat, calories and bioactive compounds with high antioxidant activity helps prevent chronic diseases and improves the health and immune system of individuals. In Mexico and Latin America, people of poor families have increased their consumption of ultraprocessed products, animal fat, proteins, sugar, high-calorie foods and products with little or no nutritional value, which leads to problems of malnutrition, overweight, obesity, diabetes and other chronic degenerative diseases [3,5], and these problems are more severe in urban, suburban and marginalized or low-income areas.

In all indigenous communities, there is local knowledge about the consumption of wild and semicultivated plants that complement or compose the basic diet of people, and in situations where it is difficult to access to basic products, these plants are used to combat hunger and malnutrition within the community [6]. In general, edible native plants are wild or semidomesticated but are easily accessible; local knowledge is needed for their identification and for information concerning their cultural heritage with respect to their use as food. Furthermore, their diversity depends on the biogeographic region of distribution. In this context, there are three crucial elements for defining the use of plants for food: local knowledge, plant diversity and plant composition as a function of the environment that expresses its properties. For example, in the Congo and Brazil, plants are extracted from forested areas [7,8], and they have certain nutritional values based on the species and origin. A similar situation occurs with respect to Mediterranean plants [9], with plants having particular flavors distributed throughout the Iberian Peninsula [10]. Other cases involve plants in cultivated areas of the Mesoamerican region, specifically in *milpa* systems [11] and home gardens [12,13].

Edible native plants, both wild and those tolerated in crop fields, are sources of macro- and micronutrients such as vitamins, minerals, fiber, amino acids and proteins, carbohydrates and functional compounds in sufficient quantities to satisfy the daily requirements of an individual [14,15,16]. However, despite the importance of these native vegetables for all indigenous communities, there are few studies on the nutritional and nutraceutical composition of their edible leaves, stems, flowers, roots and tubers. For example, in Mexico, approximately 2168 different food species, not solely quelites, have been recorded based on ethnobotanical studies of useful plants, but the proximate analysis and protein, amino acid and bioactive compound profiles are available for fewer than 30 species, including those of the genera *Amaranthus*, *Begonia*, *Chenopodium*, *Cyclanthera*, *Erythrina*, *Sechium*, *Cucurbita*, *Xanthosoma*, *Phaseolus* and *Phytolacca* [17]. Species with edible stems and leaves stand out in their supply of micronutrients to alleviate nutritional deficiencies, mainly in children and pregnant women, and prevent chronic diseases [18,19]. Guil-Guerrero [20] noted that, in addition to documenting the nutritional composition of wild plants, their cytotoxic, allergic or poisonous effects should be studied. In the present work, a review was conducted of the edible native species of Mexico called ‘quelites’; special emphasis was placed on those with edible leaves, vines and stems, and on their nutritional composition, bioactive compound content, ability to alleviate dietary deficiencies, nutraceutical potential and antimicrobial use. Additionally, the authors’ field and laboratory experiences with edible native plants used in the indigenous communities of Mexico were included.

## 2. Biogeographic Context of Plants and Comparative Analysis

This review focused on two basic principles of inclusion: (1) only the references on plants native and those originating in Mexico were included based on the reports of Villaseñor [21], excluding plants with origins in other countries [22], such as quinua from South America and many other cases, and (2) we focused on species with edible young stems, leaves, vines and flowers, which excluded species with edible grains or cereals, tubers, fruits and strawberries. In this biogeographic context, the review included all species named in Mexico as ‘quelites’ based on ethnobotanical reports and the authors’ documentation, which is explained in more detail in the next section.

An initial goal was to determine which Mexican quelites have been analyzed for their composition, and afterward, a special emphasis was also given to the phenolic compounds from green tissues and their antioxidant activity and potential effects on human health and antimicrobial action based on in vivo and in vitro evaluations. Therefore, based on an average number of 250 Mexican quelite species [17], we found that no more than 15% of the expected total had been subjects of composition and biological activity studies, as discussed below. In addition, the bioactive compounds are products of secondary metabolism (i.e., phenylpropanoid pathway), where the type and concentration of compounds are influenced by species, genotype or cultivar, environment in which the plant developed and species/genotype-environment interactions [23]. Consequently, comparisons of the composition and/or extracts of species are not feasible due to differences among species and raw materials because all evaluated species do not originate from the same growing conditions and are not separable from genetic or genotypic environmental effects. Therefore, we predominantly analyzed the experimental and biological effects as a function of the major compounds reported instead of comparing their compositions among species, but we did consider the chemical characteristics of the extracted compounds.

## 3. Diversity and Consumption of Quelites from Mexico

In Mexico, the word “*quelite*,” derived from the indigenous Nahuatl word “*quilitl*,” is used to group all herbaceous, woody, creeping or shrubby plants with or without edible flowers, leaves, vines and stems, regardless of whether they are unprocessed (fresh) or processed. These plants represent a fraction of the biodiversity of plants used as food, which includes grains, fruits, seeds, oilseeds, roots, tubers and other edible structures. Based on floristic and ethnobotanical lists, Mapes and Basurto [17] estimated that in Mexico, there are more than 250 wild, semicultivated and cultivated quelite species that grow in natural areas, are tolerated in crop fields, are on the borders of plots or home gardens or are cultivated for marketing. The main families include the *Leguminosae*, *Asteraceae*, *Begoniaceae*, *Brassicaceae*, *Solanaceae*, *Piperaceae*, *Euphorbiaceae*, *Cucurbitaceae*, *Amaranthaceae*, *Convolvulaceae*, *Chenopodiaceae* and 30 others (Table 1). However, fewer than 10 crop species occupy 12.6 million hectares cultivated in Mexico, constituting 85% of the total cultivated area [1] and reflecting a global pattern that is repeated from country to country. This indicates that the diversity of food species is underutilized, a fact that influences dietary diversity and nutritional quality with consequences such as nutritional deficiencies that arise because the main crops are essentially grain and cereal crops, not vegetable crops. Increasing dietary diversity with the consumption of a greater number of species has been proposed as an effective and sustainable strategy to eradicate micronutrient deficiency problems and malnutrition problems associated with overweight and diabetes due to the high intake of fats and ultraprocessed foods and to facilitate access to food, all through the increased consumption of quelites or traditional local vegetables [6,11,24].

Species of quelites or plants with edible stems, leaves, vines and flowers are distributed globally, and in Mexico, they are found in natural and disturbed environments, grazing areas, all agrosystems, parks and protected natural areas. They are widely used in the territories of indigenous communities, where a great number of previously unknown edible species have been recognized, although their consumption is diffused locally or regionally, and they are sold only in local and regional markets where residents are likely to recognize and use them. There is a direct relationship between local knowledge of species and how they are consumed, even when there are cultural transformations of consumption and environmental or anthropogenic changes that shift the distribution or cause the extinction of species [25]. Species of quelites are distributed in practically all eco- and agrosystems in Mexico, from the northern desert [26] to the humid tropical regions of the southern portion [27]. This fact is evident in different ethnobotanical studies that list edible plant species, although the number of species varies from site to site depending on the biogeographic context, the ecosystem, the agrosystem and the specific objectives of each study; however, together, such studies indicate the number of food species that can be considered to be members of the group of quelites. For example, in Candelaria Loxicha, Oaxaca, 73 food species were recorded, and only 16 were quelites in Sierra Norte de Puebla, 94 species were recorded, and 80 were quelites. Consequently, the number of recorded quelite species continues to increase (Table 2). Collective findings indicate that there are more than 250 species of quelites, as indicated by Mapes and Basurto [17], and this number can be estimated by considering the presence of three or four species per genus, multiplied by 84 genera, as listed in Table 1.

## 4. Composition of Edible Stems, Leaves and Flowers of Quelites from Mexico

In all indigenous and rural communities in different countries, knowledge about the consumption of wild or recently domesticated plants is among the strategies for survival and adaptation to environmental conditions. In this and other studies, efforts have been made to assess traditional local and indigenous diets through the documentation of the use of food plants with local or regional distribution, which are unknown at the national and international levels; these evaluations are based on the composition of the edible portion and its contribution to human health [7,38,39]. An additional value of traditional diets is the contribution of foods that have a variety of flavors, colors, aromas and forms of preparation with a low number of additional inputs; for example, traditional foods are often consumed directly in fresh form or with minimal processing.

### 4.1. Nutritional Composition

Species of quelites with edible leaves, flowers, vines and stems contribute a significant amount of carbohydrates, fats, dietary fiber, protein, vitamins and minerals to the diet. For example, in the genera *Amaranthus*, *Chenopodium*, *Begonia*, *Crotalaria*, *Anoda*, *Cyclanthera*, *Calandrinia*, *Porophyllum*, *Taraxacum*, *Tinantia*, *Xanthosoma*, *Lippia*, *Piper*, *Peperomia* and *Galinsoga*, the protein content varies from 2.7 to 44.2%, the fiber content reaches 22.7%, the ash content reaches 13.0% and the carbohydrate content reaches 56.8% (dry weight), with large variations within each genus and species [40,41,42,43,44,45,46,47,48,49,50,51,52,53,54]. These characteristics make the proximate composition of quelites comparable to that of conventional leafy vegetables.

In the present review, it was determined that protein quality is a function of essential amino acids in reference to the quelite species listed in Table 1. For example, when studying *Amaranthus hybridus*, Akubugwo et al. [55] discovered relevant concentrations of histidine, alanine, arginine, aspartic acid, glutamic acid, glycine, proline, serine, isoleucine, leucine, lysine, methionine, cysteine, phenylalanine, tyrosine, threonine and valine. Bressani [56] quantified different contents of valine, threonine, isoleucine, leucine, lysine, histidine and arginine in *Crotalaria longirostrata*. In these two cases, *A. hybridus* and *C. longirostrata* have six essential amino acids in concentrations that vary from 1.76 to 6.7 g/100 g protein and contain histidine, which is indispensable for children and pregnant women. From a rural community perspective, these edible plants are locally available, inexpensive protein and amino acid sources. However, it is necessary to not solely evaluate plants from Mexico [7,15].

One of the greatest contributions of edible leafy plants or quelites is their content of minerals such as K, Ca, Mg, P, S, Fe, Zn, Mn, Na, B, Mo, Cu and Co, which varies widely among the genera *Amaranthus*, *Anoda*, *Begonia*, *Calandrinia*, *Chenopodium*, *Crotalaria*, *Cucurbita*, *Cyclanthera*, *Erythrina*, *Lippia*, *Phaseolus*, *Peperomia*, *Piper*, *Porophyllum*, *Portulaca*, *Suaeda*, *Tinantia*, *Tridax* and *Xanthosoma* [32,40,43,49,53,54,57,58,59]. In the genera *Amaranthus*, *Begonia*, *Cyclanthera*, *Cucurbita* (leaves), *Phytolacca*, *Piper*, *Porophyllum*, *Solanum* and *Tinantia*, important amounts of vitamins A, B_1_, B_2_, B_3_, B_6_, E and C, as well as niacin, have been determined [35,39,43,50,55]. In addition, the leaves and stems of some edible plants are a source of pigments; for example, carotenoids are present in *Amaranthus cruentus*, *Chenopodium berlandieri* and *Portulaca oleracea* [41,54], and betalains are present in *Amaranthus hypochondriacus* [48].

### 4.2. Antioxidant Compounds

The leaves, stems and flowers of some edible plants are a source of natural antioxidant phytochemicals, such as phenolic compounds, flavonoids, alkaloids, saponins, carotenoids, betalains and chlorophyll (Table 3). For example, carotenoids are present in *Begonia nelumbiifolia* [60], and betalains are present in *Amaranthus* spp. Significant amounts of flavonoid and phenolic compound contents have been recorded in the genera *Amaranthus*, *Begonia*, *Brassica*, *Chenopodium*, *Phaseolus*, *Piper*, *Porophyllum* and *Portulaca*. Regarding specific flavonoids, *Begonia nelumbiifolia* contains quercetin, rutin and kaempferol [60], and myricetin has been identified in *Brassica rapa* [61]. In addition, Santiago-Saenz et al. [54] detected the presence of rutin, phloridzin, myricetin, quercetin and phloretin in *Chenopodium berlandieri*, and Velázquez-Ibarra et al. [51] and Santiago-Saenz et al. [54] detected a high variation in myricetin and apigenin in *Portulaca oleracea*. Regarding specific phenolic compounds, Seong et al. [61] detected the presence of caffeic acid, sinapic acid, *p*-coumaric acid and ferulic acid in *Brassica rapa*, and Santiago-Saenz et al. [54] detected the presence of caffeic, gallic, chlorogenic, vanillic, *p*-hydroxybenzoic, ferulic and syringic acids in *Chenopodium berlandieri*. In addition, in *Phaseolus coccineus*, Szymańska and Kruk [62] determined the presence of alpha- and gamma-tocopherols; in *Portulaca oleracea*, Santiago-Saenz et al. [54] identified caffeic, gallic, vanillic and ferulic acids. Additionally, tannins have been identified in *Portulaca oleracea* [51], *Chenopodium nuttalliae* [43,44,53], *Brassica rapa* [61], *Amaranthus hybridus* [55] and *Amaranthus cruentus* [41]. In addition, Barrón-Yánez et al. [43] detected the presence of saponins in *Chenopodium nuttalliae*, and Ibarra-Estrada et al. [63] identified erisovine and erisodine alkaloids in *Erythrina Americana* [64]. In *Begonia nelumbiifolia*, Villa-Ruano et al. [60] evaluated the content of oxalic acid and ascorbic acid, and in *Chenopodium berlandieri*, Lazo-Vélez et al. [65] identified triterpenoid saponins. These results indicate that there are different bioactive compounds that have not yet been evaluated and identified in various edible leafy plant species preserved by indigenous communities.

The antioxidant activity is commonly assayed by different methods, such as 1,1-diphenyl-2-picrylhydrazyl (DPPH) radical scavenging, ferric reducing antioxidant power (FRAP), oxygen radical absorbance capacity (ORAC), 2,2-azinobis 3-ethylbenzothiazoline-6-sulfonic acid radical (ABTS) scavenging and *N*,*N*-dimethyl-*p*-phenylenediamine radical (DMPD) scavenging applied to study this group of quelites (Table 3); the phytochemical compounds present have been shown to have the ability to sequester reactive species and/or stop molecular oxidation during oxidative stress [66,67].

Phenolic compounds, flavonoids, betacyanins and chlorophyll are some of the compounds responsible for antioxidant activity in quelites. Román-Cortés et al. [53] detected a significant positive correlation between total phenolic compounds and flavonoids with respect to antioxidant activity determined by ABTS, DPPH and DPMD methods. Using FRAP and ORAC, Li et al. [48] also found correlations between total phenolic compounds, total flavonoids and total betalains, and Sarker et al. [68] found correlations between total phenolic compounds, flavonoids, carotenoids, betacyanin, betaxanthin, betalain and chlorophyll via DPPH and ABTS. Although these tests estimate the groups of molecules present, they provide information on their activity or the synergism between them. However, the antioxidant activity in these types of plant can vary according to the genus [53], species [49] and accession within each species [68,69,70]. The results can also differ in each part of the plant; for example, in *Amaranthus* spp., antioxidant activity (FRAP and ORAC) is different in the seeds, stems, leaves, flowers and shoots, with the highest values found in leaves and the lowest in the seeds [48].

It should be noted that the most consumed quelites in rural populations, such as ‘huauzontles’ (*Chenopodium nuttalliae*), ‘quelites’ (*Chenopodium album*), ‘quintoniles’ (*Amaranthus hypochondriacus*), ‘romeritos’ (*Suaeda calceoliformis*) and ´verdolagas’ (*Portulaca oleracea*), have higher antioxidant activity (ABTS, DMPD and DPPH) than commercial leafy vegetables such as lettuce (*Lactuca sativa*) and spinach (*Spinacia oleracea*) [53]. Similarly, in accessions of *Gynandropsis gynandra*, *Cnidoscolus aconitifolius*, *Solanum scabrum* and *Crotalaria*, antioxidant activity (ORAC) was higher than in these vegetables [49,71]. Additionally, Jiménez-Aguilar [69] determined that *Amaranthus* spp. antioxidant activity (ORAC) was higher than that in those vegetable species [49,71]. This suggests that quelites may be an important source of potential antioxidant phytochemical compounds with health effects. It is evident that by including quelites in the diet, their contribution of bioactive compounds would be transcendental in populations with greater problems of malnutrition and health caused by poor eating habits in recent times.

### 4.3. Methods and Techniques for the Determination of Compounds

Assessing the compositions of leaves, stems and flowers of quelite species preponderantly depends on the compound characteristics. For example, in the evaluation of micro- and macrominerals, it is convenient to use atomic absorption spectrophotometry (AAS), inductively coupled plasma-optical spectrometry (ICP-OES) and ICP-mass spectrometry (ICP-MS), where the last two techniques offer major capacity to detect trace elements or very low concentrations [15,54,55,68]. The determination of amino acid contents by high-performance liquid chromatography (HPLC) is a feasible option when the objective is to compare samples or species and protein quality. UV-vis spectrophotometry is a frequent strategy to estimate the total phenols, flavonoids, carotenoids and other compounds based on standards of reference, but when the aim is to elucidate compound profiles or specific compounds, it is pertinent to apply HPLC with diode array detection (HPLC-DAD) and mass spectrometry detection, ultrahigh-performance liquid chromatography with diode array detection (UHPLC-DAD) or gas chromatography-mass spectrometry (GC-MS), Table 4. 

## 5. Antioxidant Compounds and Health

Quelites or edible leafy plants have the potential to prevent and reduce the incidence of diseases caused by oxidative stress, such as metabolic syndrome, diabetes, cancer and gastrointestinal and neurological diseases, among other diet-associated disorders [60,70,76]. Experiments have been performed to evaluate the nutraceutical potential of edible leafy plants or quelites.

### 5.1. Diabetes

Juárez-Reyes et al. [76] found that extracts of *Anoda cristata* leaves decrease glucose levels in hyperglycemic mice and that they have a positive effect on serum glucose, triglycerides and the uric acid content in mice with fructose-induced metabolic syndrome. The authors noted that the hypoglycemic effect and antioxidant activity of *A. cristata* are associated with acacetin and diosmetin. Thus, consumption of *A. cristata* is suggested to help reduce the risk of type 2 diabetes and cardiovascular diseases.

Aqueous extracts of leaves of *Portulaca oleracea* generate a decrease in serum glucose levels in diabetic mice. This effect is attributed to the increased insulin secretion in β-cells resulting from the improvement in the transport of glucose to peripheral tissues. Consequently, *P. oleracea* extract helps to prevent necrosis and atrophy of pancreatic tissue and increases blood circulation by opening blood vessels. Additionally, it reduces the levels of the proinflammatory cytokines TNF-α and IL-6, which are related to the pathogenesis of insulin resistance [77,78].

Methanolic extracts of *Cnidoscolus chayamansa* at concentrations of 70 mg/kg reduce the blood glucose concentration in mice with induced diabetes. The extracts act by stimulating functional pancreatic β-cells [79]. In this sense, aqueous extracts (2% *w*/*v*) of *C. chayamansa* show hypoglycemic activity in diabetic mice by inhibiting glucose absorption or improving sensitivity to insulin [73]. The effects of these extracts are related to the content of quercetin and catechin and are proposed to act as inhibitors of glucose absorption in intestinal cells [80]. Additionally, *C. chayamansa* extracts inhibited the growth of mutagenic cancer cells by 24% and 39% compared to that of sodium azide (NaN_3_) and 4-nitro-*O*-phenylenediamine (4-N-*O*-P) controls, respectively [75]; furthermore, the same extracts decreased low-density lipoproteins [LDLs] by 32% and triglycerides by 50% and consequently decreased hyperlipidemia [76].

### 5.2. Cancer

Barros et al. [75] pointed out that plants/young shoots of *Chenopodium ambrosioides* have high antioxidant activity attributed to their bioactive molecules, and the experimental results of antitumor activity by the use of infusion and methanolic extracts inhibited growth by 50% (GI_50_) in human cell lines (MCF-7, NCI-H460, HCT-15, HeLa, HepG2) at concentrations from 287.4 to > 400 μg/mL). The consumption of *Cnidoscolus aconitifolius* induces a protective effect against premalignant lesions (cancer) of the colon in rats by inhibiting cell proliferation and inflammation of colon lesions through a reduction in β-catenin and cyclooxygenase 2 (COX-2) activity [81]. Additionally, extracts of polysaccharides from *Portulaca oleracea* reduce sarcoma 180 transplanted into mice and have a mitogenic or comitogenic effects on splenocytes in mice with sarcomas by stimulating the proliferation of spleen lymphocytes, an immune response that improves immunity. In another experiment, it was demonstrated that these plant extracts can activate T cells to exert an immunomodulatory effect on tumors in mice [82]. In this respect, *Amaranthus cruentus* extracts inhibit tumor growth in colon cancer via cytotoxic activity related to the content of rutin, an antioxidant molecule that induces apoptosis and cell cycle arrest [83,84].

Jia-liang et al. [85] indicated that *Chenopodium ambrosioides* has anticancer activity based on the evaluation of an essential oil that inhibits the proliferation of breast cancer cells (MCF-7) and can be cytotoxic depending on the concentration and time of exposure. For these reasons, a minimum IC_50_ of 24 h is proposed. Essential oil of *C. ambrosioides* causes morphological alterations, membrane blebbing and chromatin condensation, which are typical characteristics of the apoptotic process, and induces DNA fragmentation in cancer cells, reducing the survival of malignant cells [86]. *C. ambrosioides* leaf extracts induce macrophage activation and phagocytic activity, increase cell recruitment and/or proliferation in secondary lymphoid organs (spleen, lymph node), induce positive immunomodulation in the body and induce nitric oxide (NO) production; together, these effects improve the immune system [87]. In addition, the evaluation of methanolic extracts of the inflorescences and leaves of *C. ambrosioides* determined that the extracts’ antitumor effects against colon, cervical and hepatocellular cancer in vitro is attributable to the content of flavonoids and phenolic acids, which are bioactive molecules with antioxidant capacity that counteract oxidative stress [75].

### 5.3. Obesity and Gastrointestinal Disorder

The ethanolic extracts of *Polygonum aviculare* L. have an anti-obesity effect in mice with induced obesity and fed high-fat diets. These extracts reduce the increase in body weight, increase in adipose tissue, adipocyte size and expression of lipogenic genes (PPARγ, SREBP-1c, aP2 and FAS) and consequently decrease serum levels of triglycerides, leptin and malondialdehyde (MDA) [88]. The variation in MDA levels indicates a decrease in lipid peroxidation, which could be related to the antioxidant properties of quercetin and other phenolic compounds present in the extracts [88,89].

Ethanolic extracts of *Amaranthus spinosus* inhibit gastrointestinal propulsive movements in mice via the α2-adrenergic receptor pathway. *A. spinosus* has antidiarrheal activity due to its inhibitory effect on the propulsion and secretion of fluids through the mechanism of α2-adrenoceptors and its antiulcerogenic activity against acute gastric ulcers, by which it reduces the peroxidation of lipids generated by alterations caused by ulcers. These effects are attributed in part to the presence of phenolics, tannins and flavonoids in *A. spinosus* and their ability to eliminate free radicals [90].

*Anoda cristata*, *Cnidoscolus aconitifolius* and *Crotalaria pumila* have counteract effects on *Helicobacter pylori* [91], a gram-negative bacterium responsible for triggering chronic gastritis, peptic ulcer diseases and gastric cancer [92]. Dichloromethane extracts of these three species exert an inhibitory effect on bacterial growth, with minimum inhibitory concentrations (MICs) of 62.5, 125 and 250 μg/mL, respectively, and inhibit approximately 50% of the adhesion of the bacterium to human adenocarcinoma gastric cells (AGS). The inhibitory activity against *H. pylori* is related to acacetin and diosmetin. Consequently, the extracts have potential for use in prophylactic treatments to eradicate *H. pylori* [91].

In addition to the direct consumption of quelites, the ability of fermentation processes to improve the functional activity of bioactive compounds has also been evaluated; for example, the effects of lactic acid fermentation of the juice of *Portulaca oleracea* with *Lactobacillus kunkeei* B7 have been examined [93,94]. In this case, fermentation improved both the juice’s antioxidant capacity and the bioavailability of vitamin B_2_ and increased the levels of phenolic compounds, γ-amino butyric acid and α-linalool. The results showed that the fermented juice of *P. oleracea* decreases the production of intracellular ROS, preserves the integrity of monolayers of Caco-2 cells with inflammatory stimuli and limits the production of proinflammatory mediators such as prostaglandin E2 via cyclooxygenase enzymes and nitric oxide via iNOS, IL-8 and MCP-1. As with dietary fiber, the fermented juice of *P. oleracea* has the ability to preserve the integrity of the intestinal barrier, thus counteracting intestinal inflammation and oxidative damage [95].

### 5.4. Other Disorders

The ethanolic extracts of *C. ambrosioides* have an anti-arthritic effect according to the results of an experiment with mice with arthritis induced by type II collagen antibody applied at a dose of 5 mg/kg. The results indicate that these extracts reduce the percentage of neutrophils and macrophages and the number of bone marrow cells and increase the number of lymphocytes and cells in inguinal lymph nodes. This treatment reduces the serum concentration of proinflammatory cytokines (IL-6 and TNF-α), which cause the destruction of joints; consequently, the extract of *C. ambrosioides* protects joints by delaying bone density loss and cartilage and bone deformities [95]. *C. ambrosioides* has also been studied as an antifertility or contraceptive agent. Ain et al. [96] found that methanolic leaf extracts exert antifertility effects in adult male Sprague–Dawley rats with partial sterility induced by altering the spermatogenic cycle via oxidative stress and hormone imbalance. Although additional studies are needed, these findings imply the possible use of these extracts in reproductive mechanisms, without adverse phytotoxic effects. Additionally, aqueous extracts of *C. ambrosioides* have hypotensive properties and are partially associated with cardiac effects [97], and the methanolic extracts of *C. ambrosioides* induce an endothelium-dependent vasodilator effect by stimulating muscarinic receptors, which are involved in the opening of potassium (K + Ca) channels; these effects are attributed to kaempferol, quercetin and associated compounds [98].

Studies of neurodegenerative disorders indicate that *P. oleracea* extracts have anti-apoptotic and antioxidant effects in rats with brain damage induced by rotenone, a neurotoxin. Moneim et al. [99] assessed the extracts of *P. oleracea* (1.5 mL/kg) administered to Wistar albino rats with induced brain injury with rotenone (12 mg/kg) and Parkinson-like degeneration, and they found a significant decrease in apoptotic cells in the striatum. The results are explained by immunohistochemical detection, which indicated that *P. oleracea* treatment increased the expression of B-cell lymphoma-2 (Bcl-2) and decreased the levels of nuclear factor kappa B (NF-κB) as a consequence of high synthesis of ROS and a reduction in thiobarbituric acid reactive substances, nitrite/nitrate and lactate dehydrogenase. Nevertheless, cultivated and wild forms of *P. oleracea* should be assessed in more detail, because this species has other compounds not considered in the approach, such as α-tocopherols and omega-3 acids (e.g., alpha-linoleic acid), among others [72].

The leaves and young stems of quelites have functional compounds such as phenolic acids, flavonoids (isoquercitrin, nicotiflorin, rutin, 4-hydroxybenzoic acid, syringic acid, vanillic acid), ascorbic acid, betanin, amaranthine, iso-amarathine, betacyanins and other compounds recorded previously, whose current initiatives are evaluated by extracts or infusions of quelites, assumed to be a complex of compounds, to assess their beneficial effect in preventing metabolic syndrome [83,84], cancer, rheumatoid arthritis [95], chronic inflammatory disease, systemic joint disorders leading to joint damage and structural bone damage [100,101]. Furthermore, they have prophylactic potential against brain damage and neurodegenerative diseases related to oxidative and gastrointestinal stress [90,99].

## 6. Antibacterial and Antifungal Activity of Quelite Extracts

In relation to the antifungal effects of edible leafy plants, it was experimentally demonstrated that the extracts, leaves and stems of *Amaranthus retroflexus* have antifungal effects on five fungal strains: *Penicillium verrucosum* var. *verrucosum* (NBIMCC 2003 NRRL F-143), *P. expansum*, *Fusarium graminearum* (NBIMCC 2294 IMI 155426), *Aspergillus ochraceus* (NBIMCC 2002 IM-BAS) and *A. niger* [102]; all of these fungi produce different mycotoxins that affect human health. *Galinsoga parviflora* Cav. has antibacterial and antifungal effects against *Bacillus subtilis*, *Pseudomonas aeruginosa*, *Escherichia coli*, *Aspergillus niger* and *Candida albicans* [103], and the compounds identified as probable inhibitors include caffeoyl derivatives, isoquercitrin, quercimeritrin and quercetagetin [104]. *Portulaca oleracea* has antibacterial, antifungal and antiviral activities against gram-negative strains such as *Escherichia coli*, *Pseudomonas aeruginosa* and *Neisseria gonorrhoeae*, and gram-positive strains such as *Staphylococcus aureus*, *Bacillus subtilis* and *Streptococcus faecalis*, as well as antifungal activity against *Candida albicans* [105]; additionally, *P. oleracea* has protective effects against dermatophytes of the genus *Trichophyton* [106] and displays antiherpes properties against simplex virus type 2 [107].

Crotalaria longirostrata has antibacterial effects against Escherichia coli, Citrobacter freundii and Staphylococcus epidermidis and antifungal effects against Fusarium oxysporum A. comiteca, Fusarium oxysporum A. tequilana and Fusarium solani A. comiteca. The antifungal activity of C. longirostrata is attributed to phenolic compounds, alkaloids, essential oils and glycosides [108]. Suaeda nigra shows inhibitory effects on gram-positive strains (Bacillus cereus ATCC11778, B. subtilis ATCC6633, Staphylococcus aureus ATCC29737 and Corynebacterium rubrum ATCC14898) and gram-negative bacteria (Escherichia coli NCIM2931, Pseudomonas aeruginosa ATCC9027, Salmonella typhimurium ATCC23564 and Klebsiella pneumonia NCIM2719) and has antifungal effects against Candida albicans ATCC209l, Cryptococcus neoformans ATCC34664, Candida glabrata NCIM3448 and Candida apicola NCIM3367 [109]. The dichloromethane extract of Porophyllum ruderale has antiparasitic activity against both promastigote and axenic amastigote forms of Leishmania amazonensis (leishmaniasis), with is the causal agent of a disease transmitted by mosquitoes [110]. The ethyl acetate extracts of Dysphania ambrosioides and Chenopodium ambrosioides (at concentrations ranging from 4.29 to 34.37 mg/mL) have inhibitory effects against Staphylococcus aureus, Pseudomonas aeruginosa, Enterococcus faecalis, Paenibacillus apiarius and P. thiaminolyticus, and their chloroform extracts have inhibitory effects against Mycobacterium tuberculosis, M. smegmatis and M. avium (with an MIC ranging from 156.25 to 625.0 μg/mL) attributed to the presence of rutin (12.5 ± 0.20 mg/g), ethyl acetate (16.5 ± 0.37 mg/g), and n-butanol (8.85 ± 0.11 mg/g) as well as both quercetin and chrysin in the chloroform fractions (1.95 ± 0.04 and 1.04 ± 0.01 mg/g, respectively) [74]. In Cnidoscolus aconitifolius, different compounds such as alkaloids, tannins, phlobatannins, saponins, flavonoids, cardiac glycosides, steroids and terpenoids have been identified that have antibacterial effects against Staphylococcus aureus, Pseudomonas aeruginosa, Klebsiella pneumoniae, Escherichia coli and Bacillus subtilis and antifungal effects against Aspergillus tamarii and Aspergillus niger [111,112].

Eryngium foetidum has antibacterial effects against Bacillus megaterium (MTCC 8510), B. subtilis (MTCC 441), B. flexus (MTCC 7024), Staphylococcus aureus (MTCC 96), Pseudomonas oleovorans (MTCC 617), P. aeruginosa, Klebsiella pneumoniae (MTCC 7028), Salmonella enterica (MTCC 1164) and Escherichia coli (MTCC 723) and antifungal effects against the fungus Candida albicans; these effects are attributed to different bioactive compounds, such as glycosides, flavonoids, phenols, saponins, tannins, anthocyanins, sterols, triterpenoids and anthraquinones [113,114]. E. foetidum has antimicrobial effects against six clinical strains of Helicobacter pylori, as evidenced by in vitro and in vivo assays [115].

Erythrina americana has antifungal effects against Alternaria solani, Botrytis cinerea, Fusarium oxysporum, Monilinia fructicola, Penicillium spp. and Trichoderma harzianum [63]. Similarly, Cestrum nocturnum has antifungal activity against Aspergillus fumigatus, A. versicolor, A. ochraceus, A. niger, Trichoderma viride, Penicillium funiculosum, P. ochrochloron, P. verrucosum var. cyclopium, Botrytis cinerea, Colletotrichum capsici, Fusarium oxysporum, F. solani, Phytophthora capsici, Rhizoctonia solani and Sclerotinia sclerotiorum, fungi that causes diseases in crop plants. Additionally, C. nocturnum has antibacterial activity against Enterobacter cloacae (human isolate), Escherichia coli, Klebsiella pneumonia, Proteus vulgaris, Pseudomonas aeruginosa, Salmonella paratyphi A, S. paratyphi B, S. typhimurium, Shigella flexneri, Bacillus cereus (clinical isolate), Listeria monocytogenes, Micrococcus flavus and Staphylococcus aureus; these effects are attributed to different compounds, such as carbohydrates and/or glycosides, tannins, condensed tannins, hydrolysable tannins, alkaloids and/or nitrogenous bases, flavonoids, sterols and/or triterpenes, saponins and coumarins [109].

Begonia maculata, B. soli-mutata, B. goegoensis, B. foliosa, hybrid Begonia x erythrophylla, B. thiemei, B. peltata, B. heracleifolia, B. dregei and B. mexicana have antibacterial effects against Escherichia coli (ATCC 25922), Staphylococcus aureus, Klebsiella pneumoniae, Pseudomonas aeruginosa and Citrobacter freundii; Escherichia coli is specifically inhibited by extracts of Begonia maculata leaves, and Citrobacter freundii is inhibited by extracts of Begonia thiemei and B. foliosa [116,117]. The different effects are an indicator of the different chemical compositions of the extracts or the different reactions of the pathogenic bacteria.

The extracts of the edible leaves of Persea americana are capable of inhibiting the growth of Streptococcus pyogenes, Proteus mirabilis, Klebsiella pneumoniae, Staphylococcus aureus (NCIB 8588), Klebsiella pneumoniae (NCIB 418) and Pseudomonas aeruginosa (NCIB 950), but this inhibitory effect was not observed against Salmonella typhi, Escherichia coli, E. coli (NCIB 86) or Bacillus subtilis (NCIB 3610) [118].

Edible plants have potential use in agriculture and livestock. *Phytolacca dodecandra* has natural fungicidal activity against chocolate spot caused by *Botrytis fabae*, a leaf disease of fava bean (*Vicia faba* L.) [119], and it has a bioinsecticidal effect against the larvae of ticks (*Rhipicephalus appendiculatus*), organisms that transmit diseases to cattle and cause severe losses of livestock [120]. *P. dodecandra* has antibacterial activity against *Staphylococcus aureus* ATCC 25923, *Escherichia coli* ATCC 8739 and *Pseudomonas aeruginosa* ATCC 9027 [121]. *P. dodecandra* contains various secondary metabolites, such as polyphenols, flavonoids, tannins, saponins, alkaloids, leucoanthocyanins, anthocyanins, steroids and triterpenoids, compounds to which antifungal and antibacterial properties are attributed [120,121]. *Phytolacca americana* has potential effects on periodontal inflammatory diseases and on caries caused by *Porphyromonas gingivalis* and *Streptococcus mutans* and has potential effects against *Escherichia coli*, and its effects are attributed to high concentrations of kaempferol, quercetin, quercetin 3-glucoside, isoquercitrin and ferulic acid [122]. *Phytolacca tetramera* has antifungal effects against *Colletotrichum gloeosporioides* (Penz.) Sacc., a fungus that causes losses in the production of agricultural, forestry and ornamental crop species [123].

Rumex dentatus, R. tingitanus, R. vesicarius, R. acetosella, R. acetosa, R. alpinus, R. aquaticus, R. conglomeratus, R. crispus, R. hydrolapathum, R. obtusifolius subsp. obtusifolius, R. obtusifolius subsp. subalpinus, R. patientia, R. pulcher, R. scutatus, R. stenophyllus and R. thyrsiflorus have antibacterial and antifungal activities against Shigella flexneri, Pseudomonas aeruginosa, Salmonella typhimurium, Staphylococcus epidermidis, S. aureus, methicillin-resistant S. aureus (MRSA), Bacillus subtilis catarrhalis, Streptococcus pyogenes, S. pneumoniae, S. agalactiae, Pseudomonas aeruginosa, Escherichia coli and Klebsiella pneumoniae. These plants also have a phytopathogenic effect against bacteria that cause losses in agricultural production, such as Agrobacterium tumefaciens C58, Agrobacterium tumefaciens B6, Aspergillus niger CTM 10099 and Fusarium graminearum (ISPAVE 271), and on fungi such as F. oxysporum (CTM10402), F. culmorum 21 w, Alternaria alternata (CTM 10230), Aspergillus versicolor, A. flavus, Acremonium spp., Penicillium dimorphosporum, Candida albicans, C. krusei and C. parapsilosis [124,125,126,127].

*Sechium edule* has antibacterial effects against gram-positive bacteria (*Enterococcus faecalis*, *Staphylococcus aureus*, coagulase-negative staphylococci, *S. pyogenes*, *S. agalactiae*, *S. aureus* ATCC 29213 and *Enterococcus faecalis* ATCC 29212) [128]. Similarly, *Solanum torvum* has an antibacterial effect against *Salmonella typhi* [129], and *Solanum trilobatum* inhibits the growth of *Staphylococcus aureus*, *Streptococcus pyrogens*, *Salmonella typhi*, *Pseudomonas aeruginosa*, *Proteus vulgaris* and *Escherichia coli;* in both cases, the effects are attributed to the presence of tannins [130].

According to Holetz et al. [131], the extracts of *Cestrum nocturnum*, *Eryngium foetidum*, *Persea americana*, *Porophyllum ruderale*, *Sechium edule*, and *Solanum torvum* are classified as having good antimicrobial activity (MIC less than 100 μg/mL; Table 5), while the extracts of *Phytolacca americana* were moderate (IMC from 100 to 500 μg/mL), and the extracts of *Solanum trilobatum*, *Dysphania ambrosioides* and *Chenopodium ambrosioides* were considered inactive (IMC greater than 1000 μg/mL). Most quelites displayed higher antimicrobial activity than did the most consumed vegetables, as was the case for the following: *Lactuca sativa* (IMC of 2500 μg/mL) against *Escherichia coli* (ATCC25922), *Klebsiella pneumoniae* CI, *Staphylococcus aureus* and *Bacillus subtilis* ATCC 6633 [132]; *Spinacia oleracea* (IMC of 60,000–70,000 μg/mL) against *S. aureus* (ATCC 29213) and *E. coli* OH157:H7 (ATCC 25922) [133]; and *Brassica oleracea* (IMC of 2500–20,000 μg/mL) against *E. coli* O157:H7 ATCC 35150, *Salmonella typhimurium* ATCC 14028, *Listeria monocytogenes* ATCC 35152, *B. cereus* ATCC 11778 and *S. aureus* [134].

## 7. Remarks and Perspectives

The efforts and natural and economic resources of the global production and marketing of food have focused on a few species or products, which has led to an increase in diet homogenization, with nutritional consequences and repercussions on health, generating another source of malnutrition in addition to that caused by dietary changes or transformations. Consumption patterns indicate that there is reduced consumption of vegetables [5] and that a small number of leafy plant species, which can provide high levels of nutrients, are marketed. In contrast, among indigenous communities, a large number of plant species with edible stems, leaves, vines and flowers are consumed and locally marketed. In Mexico, these plants are known as quelites or *quilitl* in the Nahuatl language, although this group of plants also takes other names in indigenous native languages and comprises more than 250 species. However, in Mexico, no more than 10 domesticated species occupy most of the cultivated area, corresponding to 12.6 million hectares (FAOSTAT, 2019) [1]. The inclusion of quelites in people’s diet depends on documented knowledge of their nutritional and nutraceutical potential. The most studied genera with species native to Mexico are *Amaranthus*, *Chenopodium*, *Begonia*, *Crotalaria*, *Anoda*, *Cyclanthera*, *Calandrinia*, *Porophyllum*, *Taraxacum*, *Tinantia*, *Xanthosoma*, *Lippia*, *Piper*, *Peperomia*, *Cucurbita* and *Galinsoga*. Quelites provide proteins, amino acids, minerals, vitamins A, B complex, C and E and a significant amount of bioactive compounds, such as carotenoids, betalains, flavonoids, phenolic compounds and, in some cases, alkaloids and condensed tannins, among other nutrients.

In terms of nutrients, quelite species provide 2.7% to 44.2% protein based on dry weight (50 g, dietary reference intake (DRI)) and all essential amino acids except tryptophan in quantities that vary from 1.76 to 6.7 g/100 g protein (DRI, 23–33 mg/g protein), including histidine, which is indispensable for children and pregnant women. Quelites also provide micro- and macroelements; for example, 100 g of raw *Amaranthus* spp. provides 13.6 and 3.8 mg of Fe and Zn, respectively, a sufficient amount of Fe (DRI of 10 mg/day) and 25.3% of the total required Zn (15 mg/day, DRI); both of these elements are deficient in people of poor families with low income. Additionally, quelites contain K, Ca, Mg, P, S, Mn, Na, B, Mo, Cu and Co as well as substantial amounts of vitamins A, B_1_, B_2_, B_3_, B_6_, E, and C, plus niacin.

Members of the *Amaranthus*, *Begonia*, *Brassica*, *Chenopodium*, *Phaseolus*, *Piper*, *Porophyllum* and *Portulaca* quelite genera contain bioactive compounds such as rutin, myricetin, quercetin, catechin, amaranthine, kaempferol, epigenin, *p*-coumaric acid, sinapic acid, ferulic acid, gallic acid, caffeic acid, vanillic acid, ascorbic acid, oxalic acid and syringic acid, among others, which are significantly correlated with antioxidant activity, as revealed FRAP, ORAC, ABS and DMPD assays. In addition, in *Chenopodium* spp., *Erythrina americana* and *Portulaca oleracea* were identified as containing saponins, alkaloids and tannins, respectively.

In this review, many experimental assays provided evidence of the potential effects of quelite extracts to prevent, manage and probably control current human health problems, encouraging various theories and explanations of the effects. In this sense, the extracts of *Anoda cristata*, *Portulaca oleracea* and *Cnidoscolus chayamansa* present hypoglycemic activity to prevent diabetes. In addition, *Chenopodium ambrosioides*, *Cnidoscolus aconitifolius*, *Portulaca oleracea* and *Amaranthus cruentus* extracts have antitumor activity and improve the immune system. *Polygonum aviculare* extracts caused a decrease in lipid peroxidation, and *Amaranthus spinosus* extracts have antidiarrheal activity; both of these extracts have been proposed to prevent obesity. *Anoda cristata*, *Cnidoscolus aconitifolius* and *Crotalaria pumila* extracts displayed experimental anti-*Helicobacter pylori* effects.

In the antibacterial and antifungal assays and based on an MIC < 100 μg/mL, the extracts with good antimicrobial activity were those of *Suaeda nigra*, *Eryngium foetidum*, *Cestrum nocturnum* and *Solanum torvum* against *Staphylococcus*, *Enterococcus*, *Bacillus*, *Escherichia*, *Pseudomonas*, *Salmonella* and *Candida* strains. In addition, *Porophyllum ruderale* and *Persea americana* extracts inhibited the growth of *Leishmania amazonensis* and *Streptococcus* strains, respectively (Table 5).

Lastly, there were a few documented cases on the use of fermented plants, plant parts or plant juices of quelite species. For example, Filannino et al. [91] and Di cagno et al. [92] found that lactic acid fermentation of the juice of *Portulaca oleracea* improved the bioavailability of bioactive compounds and enhanced functional activity. This finding opens other possibilities of the use of other vegetable species to concoct beverages.

## Figures and Tables

**Table 1:** List of plant genera with flowers, stems or leaves frequently consumed in Mexico.

Plant Genera	Plant Genera	Plant Genera	Plant Genera
*Agave* spp.	*Cucurbita* spp.	*Lilaeopsis* spp.	*Portulaca* spp.
*Amaranthus* spp.	*Cyclanthera* spp.	*Lippia* spp.	*Quercus* spp.
*Anoda* spp.	*Dhalia* spp.	*Lobelia* spp.	*Rauwolfia* spp.
*Arthrostema* spp.	*Diastatea* spp.	*Lycianthes* spp.	*Rumex* spp.
*Arbutus* spp.	*Diphysa* spp.	*Microsechium* spp.	*Sechium* spp.
*Attalea* spp.	*Disocactus* spp.	*Mimulus* spp.	*Senecio* spp.
*Bacharis* spp.	*Dysphania* spp.	*Mosntera* spp.	*Sicyos* spp.
*Begonia* spp.	*Erblichia* spp.	*Opuntia* spp.	*Singonium* spp.
*Berula* spp.	*Erygium* spp.	*Orbignya* spp.	*Smilax* spp.
*Bidens* spp.	*Erythrina* spp.	*Oxalis* spp.	*Solanum* spp.
*Brassica* spp.	*Eupatorium* spp.	*Ouratea* spp.	*Sonchus* spp.
*Calindrinia* spp	*Galinsoga* spp.	*Peperomia* spp.	*Spergula* spp.
*Cestrum* spp.	*Hydrocotyle* spp.	*Persea* spp.	*Stellaria* spp.
*Cichorium* spp.	*Hylocereus* spp.	*Petroselinum* spp.	*Suaeda* spp.
*Chamaedorea* spp.	*Ipomea* spp.	*Phacelia* spp.	*Taraxacum* spp.
*Chenopodium* spp.	*Jaltomata* spp.	*Phaseolus* spp.	*Tauschia* spp.
*Claytonia* spp.	*Lantana* spp.	*Phytolacca* spp.	*Tinantia* spp.
*Cnidoscolus* spp.	*Lepidium* spp.	*Pilea* spp.	*Tridax* spp.
*Cotula* spp.	*Leucaena* spp.	*Piper* spp.	*Urtica* spp.
*Crysophila* spp.	*Lycianthes* spp	*Poeppigia* spp.	*Xanthosoma* spp.
*Crotalaria* spp.	*Lilaea* spp.	*Porophyllum* spp.	*Yucca* spp.

Source: Field notes complemented with Aguilar-Støen et al. [12], Balcázar-Quiñones et al. [13] and Mapes and Basurto [17].

**Table 2:** A brief list of ethnobotany studies on edible native plants in different regions from Mexico.

Region, Municipalities or States of Mexico	Food Species	Ref.
Isidro Fabela, Estado de México and Mariano Escobedo, Veracruz	12 (11 ^1^)	[28]
Candelaria Loxicha, Oaxaca	73 (16)	[12]
Amatenango del Valle, Chiapas	13 (11)	[29]
Lacanja Chansayab, Chiapas	26 (5)	[27]
Sierra Norte de Puebla	94 (80)	[30]
San Pedro Arriba, Temoaya, Estado de México	68 (49)	[13]
Ixhuapan, Meyacapan, Veracruz	69 (48)	[31]
Santa María Ixcatlán, Oaxaca	138 (ns)	[32]
Rayones, Nuevo León	83 (ns)	[33]
San Lucas Huajotitlán y Buenavista de Juárez, Chietla, Puebla	28 (ns)	[34]
59 communities of Chiapas	71 (ns)	[35]
Tilzopotla, Puente de Ixtla, Morelos	61 (ns)	[36]
San Agustín Loxicha, Candelaría Loxicha and Pluma Hidalgo, Oaxaca	131 (ns)	[37]

^1^ Species number with edible stems, leaves and/or flowers and ns = not specified.

**Table 3:** Compound involved in the secondary metabolism of quelite species and their antioxidant activity.

Species	Plant Part	Antioxidant Compounds	Antioxidant Activity ^1^	Ref.
*Amaranthus cruentus*	Whole plant	Total phenolics (gallic acid), total flavonoids (catechin) and tannins		[41]
*A. hypochondriacus*, *A. caudatus* and *A. cruentus*	Leaves, stalks, flowers, sprouts and seeds	Betalain; amaranthine; iso-amaranthine; betanin; iso-betanin-gallic, protocatechuic, chlorogenic, gentistic, 2,4-dihydroxybenzoic, ferulic, ellagic and salicylic acids; rutin; kaempferol-3-rutinoside; and quercetin	FRAP and ORAC	[48]
*A. hybridus*	Leaves	Alkaloids, flavonoids, saponins, tannins, phenols, hydrocyanic acid and phytic acid		[55]
*A. spinosus* and *A. viridis*	Leaves	Chlorophyll, total carotenoids, β-cyanin and β-xanthin content, β-carotene, vitamin C, total polyphenols and total flavonoids	DPPH and ABTS	[68]
*A. acanthochiton*, *A. deflexus* and *A. viridis*	Leaves	Vitamin C, total phenolics (gallic acid), flavonoids (catechin)	ORAC	[69]
*A. hybridus*, *Chenopodium berlandieri* and *Portulaca oleracea*	Leaves	Chlorophyll; carotenoids; ferulic, chlorogenic, caffeic, gallic, chlorogenic, vanillic, *p*-hydroxybenzoic, *p*-coumaric and syringic acids; rutin; phloridzin; myricetin; quercetin; naringenin; phloretin; galangin; apigenin	DPPH, ABTS and FRAP	[54]
*Portulaca oleracea*, *Chenopodium* spp., *Amaranthus* spp., *Chenopodium* spp. and *Suaeda* spp.	Leaves	Total phenolics (gallic acid), total flavonoids (quercetin) and condensed tannins	DPPH, ABTS and DMPD	[53]
*Cnidoscolus aconitifolius*, *Solanum scabrum*, *Crotalaria longirostrata* and *Gynandropsis gynandra*	Leaves	Vitamin C, total phenolics (gallic acid) and total flavonoids (catechin)	ORAC	[49]
*Begonia nelumbiifolia*	Stalks and leaves	β-Carotene, lutein, β-cryptoxanthin, α-carotene, quercetin, rutin and kaempferol	DPPH	[60]
*Brassica rapa*	Leaves	Caffeic acid, sinapic acid, *p*-coumaric acid, ferulic acid and myricetin	DPPH, FRAP, TEAC and ORAC	[61]
*Phaseolus coccineous*	Young leaves	α-Tocopherols, γ-tocopherols and δ- tocopherols		[62]
*Portulaca oleracea*	Whole plants	Total phenolics (gallic acid), total flavonoids (rutin) and total carotenoids	DPPH and FRAP	[70]
*Piper auritum*	Leaves	β-Pinene, α-terpinene, trans-β-ocimene, terpinolene, safrole, β-caryophyllene, germacrene D, trans-nerolidol and phytol	ABTS	[52]

^1^ DPPH, 1,1-Diphenyl-2-picrylhydrazyl; FRAP, ferric reducing antioxidant power; ORAC, oxygen radical absorbance capacity; TEAC, Trolox equivalent antioxidant capacity; DMPD, *N*,*N*-dimethyl-*p*-phenylenediamine.

**Table 4:** A list of methods and techniques used for determinate the composition in quelite species.

Compounds	Methods of Determination/Identification	Species Evaluated	Ref.
Minerals	Atomic absorption spectrophotometry (AAS)	*A. hybridus, A. viridis. A. spinosus, C. chaymansa, C. berlandieri, P. oleracea*	[15,54,55,68,70]
	Inductively coupled plasma-optical emission spectrometry (ICP-OES) and ICP-mass spectrometry (ICP-MS)	*P. oleracea, C. chaymansa, C. berlandieri, C. acnotifolius, C. longirostrata.*	[49,64,72]
Amino acids	High-performance liquid chromatography (HPLC)	*P. oleracea*	[72]
Vitamin A, C, α-tocopherol, phenols, carotenoids, flavonoids, betalains, betacyanins, saponins	UV-vis spectrophotometry and HPLC-DAD/HPLC-MS	*P. oleracea, C. chaymansa, B. nelumbiifolia, A. cruentus, A. hypochondriacus, A. caudatus, A. viridis, A. spinosus, A. hybridus, C. berlandieri, C. lingirostrata, C. nuttallie*	[15,47,48,49,54,60,65,68,70,72,73]
Phenolic compounds and profiles	High-performance liquid chromatography with diode array detection (HPLC-DAD), ultrahigh-performance liquid chromatography with diode array detection (UHPLC-DAD) and gas chromatography-mass spectrometry (GC-MS)	*C. ambrosioides, C. chaymansa, A. viridis, A. cruentus, A. hypochondriacus, A. caudatus*	[47,55,73,74]
Organic acids	Ultrafast liquid chromatography coupled to a photodiode array detector (UFLC-PDA) and HPLC-DAD	*C. ambrosioides, B. nelumbiifolia*	[60,75]

**Table 5:** Antibacterial and antifungal activities based on extracts of quelite species.

Quelite Species	Extract ^1^ (Plant Part)	Identified Compounds ^2^	Microorganisms	MIC ^3^ (μg/mL)	Ref.
*Amaranthus retroflexus*	EE and ME (whole plants)	NE	*Penicillium verrucosum* var. *verrucosum* (NBIMCC 2003 NRRL F-143), *P. expansum*, *Fusarium graminearum* (NBIMCC 2294 IMI 155426) and *Aspergillus ochraceus* (NBIMCC 2002 IM-BAS) and *A. niger*	125,000	[101]
*Galinsoga parviflora*	EE (whole plants)	Caffeoyl derivatives, isoquercitrin, quercimeritrin and quercetagetin	*Bacillus subtilis* (6633), *Pseudomonas aeruginosa* (27853), *Escherichia coli* (10538), *Aspergillus niger* (16404) and *Candida albicans* (10231)	100,000	[102]
*Portulaca oleracea*	ME (whole plants)	Lupeol, β-sitosterol and daucosterol	*Escherichia coli*, *Pseudomonas aeruginosa*, *Neisseria gonorrhea*, *Staphylococcus aureus*, *Bacillus subtilis*, and *Streptococcus faecalis* and *Candida albicans*	1000	[104]
*Crotalaria longirostrata*	PF, EAF, AF, DMF, EEF and SDW (leaves)	Phenolic compounds, alkaloids, essential oils and glycosides	*Escherichia coli* (ITTG-1879), *Citrobacter freundii* (Cf-ITTG) and *Staphylococcus epidermidis* (ITTG-850). *Fusarium oxysporum* A. Comiteca (FoC-ITTG), *Fusarium oxysporum* A. tequilana (FoT-ITTG) and *Fusarium solani* A. comiteca (FsC-ITTG).	200,000	[107]
*Suaeda nigra*	PEE, EAE, ACE, ME and AE (leaves)	Cardiac glycosides	*Bacillus cereus* (ATCC11778), *Staphylococcus aureus* (ATCC29737), *Corynebacterium rubrum* (ATCC14898), *Escherichia coli* (NCIM2931), *Pseudomonas aeruginosa* (ATCC9027), *Salmonella typhimurium* (ATCC23564), and *Klebsiella pneumonia* (NCIM2719) *Candida albicans* (ATCC209l), *Cryptococcus neoformans* (ATCC34664), *Candida glabrata* (NCIM3448) and *Candida apicola* (NCIM3367)	20	[108]
*Porophyllum ruderale*	DE (leaves)	Thiophene derivatives: 5-methyl-2,2′:5′,2″-terthiophene and 5′-methyl–[5–(4–acetoxy-1–butynyl)]–2,2′-bisthiophene	*Leishmania amazonensis* (WHOM/BR/75/JOSEFA)	60.3–77.7	[109]
*Dysphania ambrosioides* and *Chenopodium ambrosioides*	EE, CF, EAF and BF (leaves)	Rutin, quercetin and chrysin	*Staphylococcus aureus* (ATCC 25923), *Pseudomonas aeruginosa* (ATCC 340), *Enterococcus faecalis* (ATCC 29212), *Paenibacillus apiaries*, *P. thiaminolyticus*, *Mycobacterium tuberculosis* (ATCC 25618), *M*. *smegmatis* (ATCC 700084) and *M. avium* (LR541CDC)	4300–68,800	[110]
*Cnidoscolus aconitifolius*	EE (roots, leaves, and stems)	Steroids, tannins, alkaloids, cardiac glycosides, terpenoids and saponins	*Klebsiella oxytoca*, *Escherichia coli*, *Proteus species*, *Bacillus subtilis* and *Pseudomonas aeruginosa*	20,000	[111]
	ME (leaves)	Alkaloids, tannins, saponin, flavonoids and cardiac glycoside	Bacteria: *Staphylococcus aureus*, *Pseudomonas aeruginosa*, *Klebsiella pneumoniae* and *Escherichia coli* Fungi: *Aspergillus tamarii* and *Aspergillus niger*	31,250–500,000	[112]
*Eryngium foetidum*	EE, ME and AE (leaves)	Flavonoids, phenols, saponins and tannins	*Bacillus megaterium* (MTCC 8510), *Bacillus subtilis* (MTCC 441), *Bacillus flexus* (MTCC 7024), *Staphylococcus aureus* (MTCC 96), *Pseudomonas oleovorans* (MTCC 617), *Klebsiella pneumoniae* (MTCC 7028), *Salmonella enteric* (MTCC 1164) and *Escherichia coli* (MTCC 723)	25	[113]
	PEE, CE, EAE, ME and AE (leaves)	Anthocyanins, sterols, triterpenoids and anthraquinones	(a) *Escherichia coli*, *Pseudomonas aeruginosa*, *Bacillus subtilis* and *Staphylococcus aureus.* (b) *Candida albicans*	(a) 3.12–200 (b) 1.56–12.5	[114]
	ME and AE (leaves)	Alkaloids, phenols, flavonoids, anthraquinones and sterols	*Helicobacter pylori*	64–1024	[115]
*Erythrina americana*	ME (seeds)	Alkaloids	*Alternaria solani*, *Botrytis cinerea*, *Fusarium oxysporum*, *Monilinia fructicola*, *Penicillium* sp. and *Trichoderma harzianum*	NR	[63]
*Cestrum nocturnum*	CF, EAF and AF (leaves)	Triterpenes, coumarins, flavonoids, tannins, saponins and carbohydrates	(a) *Enterobacter cloacae*, *Escherichia coli* (ATCC 35210), *Pseudomonas aeruginosa* (ATCC 27853), *Salmonella typhimurium* (ATCC 13311), *Bacillus cereus* (clinical isolate), *Listeria monocytogenes* (NCTC 7973), *Micrococcus flavus* (ATCC 10240) and *Staphylococcus aureus* (ATCC 6538). (b) Fungi: *Aspergillus fumigatus* (ATCC 1022), *Aspergillus versicolor* (ATCC 11730), *Aspergillus ochraceus* (ATCC 12066), *Aspergillus niger* (ATCC 6275), *Trichoderma viride* (IAM 5061), *Penicillium funiculosum* (ATCC 36839), *Penicillium ochrochloron* (ATCC 9112) and *Penicillium verrucosum* var. *cyclopium*	(a) 0.6–3.75 (b) 0.075–2.5	[135]
	EAE, ME, EE and CE (leaves and stems)	Ethyl	*Escherichia coli*, *Klebsiella pneumonia*, *Proteus vulgaris*, *Pseudomonas aeruginosa*, *Salmonella paratyphi A*, *Salmonella paratyphi B*, *Shigella flexneri* and *Staphylococcus aureus*	55–350 and 25–400	[136]
	HE, CE, EAE and ME (flower)	Acetate, ethanol, and methanol extracts	*F. oxysporum* (KACC 41083), *Phytophthora capsici* (KACC 40157), *C. capsici* (KACC 410978), *F. solani* (KACC 41092), *Rhizoctonia solani* (KACC 40111), *S. sclerotiorum* (KACC 41065) and *B. cinerea* (KACC 40573)	125–1000	[137]
*Begonia maculata*	ME (leaves)	NE	*Escherichia coli* (ATCC 25922), *Klebsiella pneumoniae*, *Pseudomonas aeruginosa* and *Staphylococcus aureus*	20,000	[116]
*Begonia soli-mutata*, *B. goegoensis*, *B. foliosa*, *B. erythrophylla*, *B. thiemei*, *B. peltata*, *B. heracleifolia B. dregei*, *B. mexicana*	EE (leaves)	Phenol, tannins, xanthoproteins, steroids, phytosterols, triterpenoids, sapogenins, coumarins and carbohydrates	*Citrobacter freundii*	52,631	[117]
*Persea americana*	ME (leaves and bark)	Saponins, tannins, phlobatannins, anthraquinone, flavonoids and terpenoids	(a) *Bacillus subtilis* (NCIB 3610), *Staphylococcus aureus* (NCIB 8588), *Escherichia coli* (NCIB 86), *Klebsiella pneumoniae* (NCIB 418) and *Pseudomonas aeruginosa* (NCIB 950); (b) *Streptococcus pyogenes*, *Proteus mirabilis*, *Salmonella typhi*, *Klebsiella pneumoniae* and *Escherichia coli*	(a) 10,000–30,000 (b) 3–12	[118]
*Phytolacca americana*	ME, HF, CF, EF and BF	Kaempferol, quercetin, quercetin 3-glucoside, isoquercitrin and ferulic acid	*Porphyromonas gingivalis* (ATCC BAA-1703), *Streptococcus mutans* (ATCC 700610) and *Escherichia coli* (DH5α)	200–1800	[122]
*Sechium edule*	EE (leaves, stems and fruit)	Flavonoids	*Staphylococcus aureus* (ATCC 29213), *Enterococcus faecalis* (ATCC 29212), *Streptococcus agalactiae* and *Streptococcus pyogenes*	4.16–16.64 or 8.32–16.64	[128]
*Solanum torvum*	CE and ME (roots, leaves and stems)	Tannins	*Bacillus cereus*, *B. subtilis*, *Streptococcus-β-haemolyticus*, *Salmonella typhi* and *Shigella dysenteriae*	64–128	[129]

^1^ Hexane extract (HE); chloroform extract (CE); dichloromethane extract (DE); ethyl acetate extract (EAE); ethanolic extract (EE); methanol extract (ME); aqueous extract (AE); ether extract (ETE); petroleum ether extract (PEE); acetone extract (ACE); hexane fraction (HF); butanol fraction (BF); chloroform fraction (CF); ethyl acetate fraction (EAF); propanone fraction (PF); ethyl acetate fraction (EAF); aqueous fraction (AF); dichloromethane fraction (DF); ethyl ether fraction (EEF); sterile distilled water (SDW); ^2^ NE = not evaluated; ^3^ NR = not reported.
